# Clinically relevant stabilizers of the posteromedial and posterolateral knee: normal anatomy, scanning technique, and ultrasound findings in patients with anterior cruciate ligament tear

**DOI:** 10.1007/s00330-025-11868-8

**Published:** 2025-07-29

**Authors:** Riccardo Picasso, Giovanni Marcenaro, Federico Zaottini, Federico Pistoia, Marta Macciò, Ellert Jan Barendrecht, Emanuele Quarto, Maribel Miguel Pérez, Carlo Martinoli

**Affiliations:** 1 IRCCS Ospedale Policlinico San Martino, Genova, Italy; 2 Department of Health Sciences (DISSAL), Università di Genova, Genova, Italy; 3 het FysioHuis, Bergmanclinics, The Netherlands; 4 Unidad de Anatomía y Embriología Humana, Departamento de Patología y Terapéutica Experimental, Facultad de Medicina y Ciencias de la Salud (Campus de Bellvitge), Universitat de Barcelona, Barcelona, Spain

**Keywords:** Ultrasound, Anterior cruciate ligament, Posteromedial corner, Posterolateral corner

## Abstract

**Abstract:**

Unrecognized and untreated injuries of the posteromedial and posterolateral corners of the knee are more common than previously thought and have been linked to poor outcomes after anterior cruciate ligament reconstruction. Amongst imaging modalities, magnetic resonance is currently referred to as the gold standard for the evaluation of these regions, but has several limitations, in particular in the identification of subacute and chronic lesions. Recent technological advancements and the progressive refinement of linear probes have expanded the potential of High-resolution ultrasound in demonstrating the stabilizers of the posteromedial and posterolateral corners in both normal and pathological cases, and now this modality may be considered as a useful complementary tool for the evaluation of these structures. The aim of this work is twofold: (i) to review, also with the support of dedicated dissections and schematic drawings, the normal anatomy and the biomechanical role of the clinically relevant stabilizers of the posteromedial and posterolateral knee, including the distal expansion of the semimembranosus tendon, the popliteus muscle–tendon unit, and the posterior oblique ligament; (ii) to illustrate the normal ultrasound appearance of these structures and the spectrum of pathological findings that this modality may disclose in patients with anterior cruciate ligament tear.

**Key Points:**

***Question*** *Injuries of the posteromedial and posterolateral knee worsen the outcome after anterior cruciate ligament reconstruction but are often underdiagnosed on imaging*.

***Findings*** *Ultrasound has potential in diagnosing tears of stabilizers of the posteromedial and posterolateral knee and provides complementary information to MRI about ligament status and continuity*.

***Clinical relevance*** *In subacute and chronic injuries, ultrasound has some advantages over MRI as it can disclose subtle abnormalities that might otherwise be unrecognized, thereby improving diagnostic confidence and patient counselling*.

## Introduction

Anterior cruciate ligament (ACL) tears are amongst the commonest sports injuries and are associated in up to 86% of cases with the development of knee instability [1]. The refinement of surgical techniques is leading to an increase in reconstructive procedures, which are now performed in around 75% of people with ACL tears [2]. However, recent research failed to demonstrate a superiority of surgery over conservative management in improving outcomes like the development of osteoarthritis or daily living activities, suggesting that the prognosis may depend not only on the surgical technique, but also on the concomitant damage to other knee stabilizers [3–5]. In this regard, increasing attention has been devoted to the potential role of unrecognized damage to minor but functionally relevant ligaments of the posteromedial (PMC) and posterolateral (PLC) corners that, if untreated, may worsen the outcome after ACL reconstruction [6–8]. The aim of this work is to review the anatomy of the clinically relevant stabilizers of PMC and PLC and to illustrate the potential of ultrasound (US) in disclosing injuries in patients with ACL tears.

### Imaging techniques

Magnetic resonance imaging (MRI) has a recognized role in the diagnostic workup of patients with suspected PMC/PLC injury, as it can provide critical data on the status of knee stabilizers and may guide therapeutic decisions [9]. On the contrary, the role of US in diagnosing PMC and PLC tears has been largely disregarded, on one side due to the long learning curve that is necessary to master this modality, and on the other because of previous technical limitations of US in demonstrating small and deep-seated structures. However, technological advancements in US equipment now allow a comprehensive evaluation of all the relevant stabilizers of PMC and PLC and open a perspective for a potential use of this modality in the diagnostic workup of suspected injuries. Recently, the sonographic anatomy of the posteromedial and posterolateral knee has been described, and preliminary works on the potential of US in disclosing traumatic injuries have been published, even if limited literature is currently available on the diagnostic accuracy of this modality in evaluating these regions [10–12]. Of note, US may provide complementary information on knee stability through the evaluation of PMC and PLC stabilizers during stress tests and may disclose subtle findings that are indicative of PMC and PLC damage that are not readily evident on MRI. These capabilities are of particular value in the diagnosis of subacute and chronic trauma, where the sensitivity of MRI in diagnosing PMC and PLC injuries has been reported as low as 48% with respect to clinical examination [13, 14]. In these patients, US may provide a correct diagnosis by comparing the affected and the contralateral knee and disclosing minor ligament abnormalities, including asymmetrical thickening, hypoechogenity, and loss of fibrillar pattern, that may be easily missed on MRI.

### Clinically relevant stabilizers of the posteromedial corner (PMC)

PMC is located between the medial collateral ligament (MCL) and the posterior cruciate ligament (PCL) and includes the semimembranosus tendon (SMt), the posterior oblique ligament (POL), the oblique popliteal ligament (OPL), the posterior horn of the medial meniscus, and the medial head of the gastrocnemius (Table 1). Insufficiency of these structures may lead to anteromedial rotatory instability (AMRI), which consists of an increased anterior dislocation and external rotation of the medial tibial plateau with respect to the femur, associated with valgus instability of the medial joint space [15]. PMC injuries are not uncommon and may occur in severe trauma in association with ACL, PCL, and MCL tears, where the sudden contraction of SMt aimed at resisting anterior tibial translation may cause a tear of the tendon itself or strain of POL, OPL, and the joint capsule [16]. In a recent work three major patterns of PMC damage were described, consisting of (i) POL injury and strain of the capsular arm of SMt (70%); (ii) POL injury and peripheral meniscal detachment (30%), and (iii) POL injury, disruption of SMt, and peripheral meniscal detachment (19%) [17]. Management of PMC injuries depends on the extent of damage. In severe trauma, POL, posteromedial capsule, and SMt are all possible targets of surgical repair [18, 19]. In this context, imaging may have a substantial role in providing a precise description of the damaged structures, assisting the preoperative evaluation of patients, and reducing the risk of unsatisfactory postsurgical outcomes [18, 19].

**Table 1:** PMC stabilizers

	Normal US appearance	Pathological findings
SMt	Six distinct expansions inserting into the tibia, the meniscus, the POL, and the OPL	Discontinuity, detachment, and bone avulsion in case of complete tear; thickening, hypoechogenicity, and inhomogeneous echotexture in case of partial tears
POL	Hyperechoic and fibrillar thickening of the posteromedial tibiofemoral joint capsule	Thickening, wavy appearance, and inhomogeneous hypoechoic echotexture, often associated with SMt tear
OPL	Transversely oriented hyperechoic and fibrillar ligament running on the posterior aspect of the tibiofemoral joint capsule. Possible anisotropy of the medial third	Thickening and hypoechogenicity

### The SMt

**Fig. 1:**
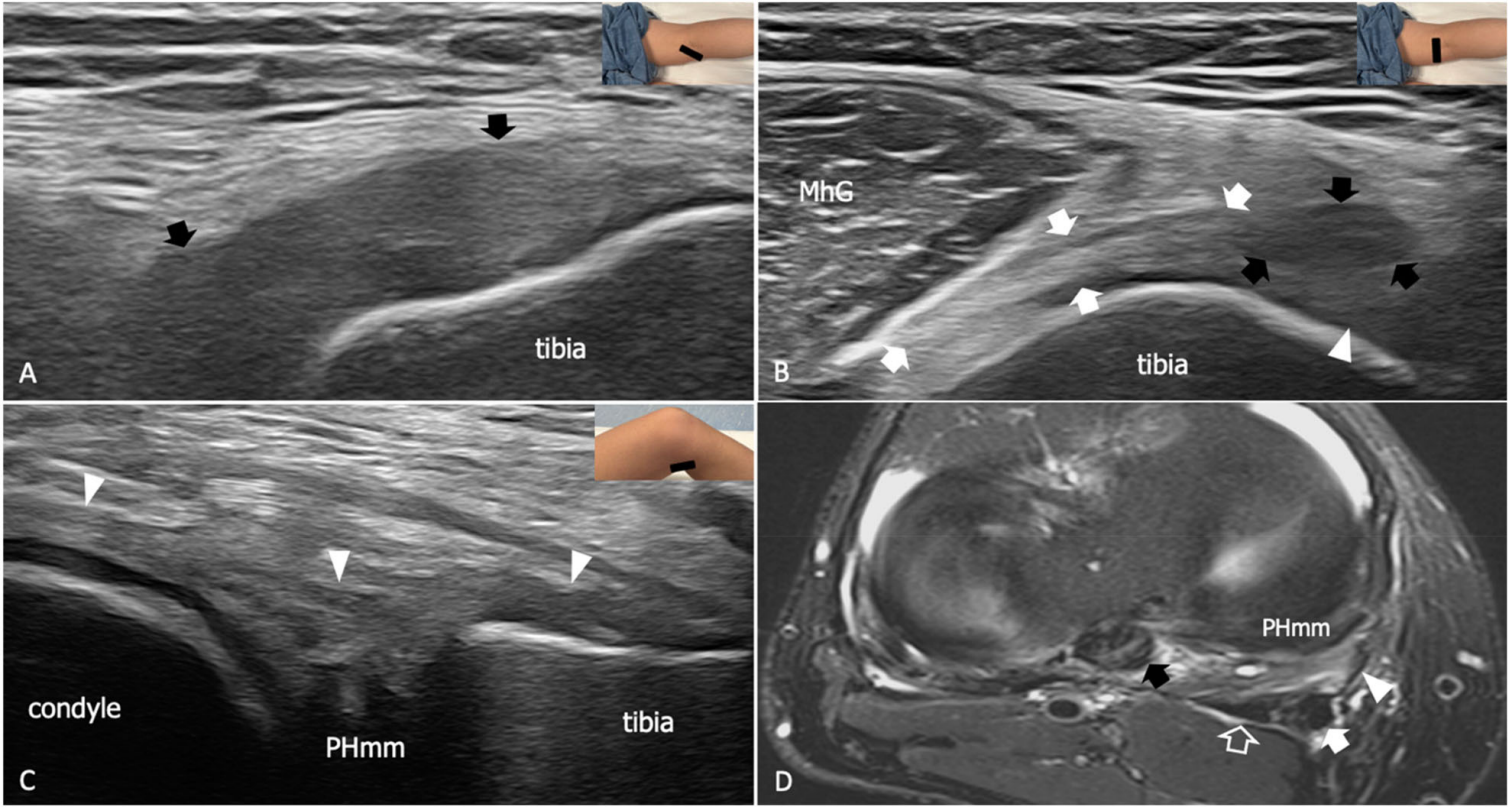
Sprain of the anterior arm of SMt in a 34-year-old football player with anterior and posterior cruciate ligaments. **A** Long-axis 18-5 MHz US image demonstrates an abnormally thickened and hypoechoic anterior arm of SMt (black arrows). **B** Short-axis 18-5 MHz US scan illustrates the different echotexture of the regularly fibrillar and hyperechoic direct arm (white arrows) and the thickened and hypoechoic anterior arm (black arrows), the latter running over the tibial arm of POL (arrowhead). **C** Long-axis 18-5 MHz US scan shows an associated distraction of the tibial arm of POL (arrowheads), which appears thickened and hypoechoic with irregular margins. **D** Comparative axial tSE T2-weighted 3 T scan with fat saturation confirms the distraction of the anterior arm of SMt (white arrow) and of the tibial arm of POL (arrowhead), as well as the normal appearance of the direct arm (outlined arrow). Note the abnormal aspect of the posterior cruciate ligament (black arrow). MhG, medial head of the gastrocnemius; PHmm, posterior horn of the medial meniscus. The inserts illustrate the respective probe position

SMt plays a pivotal role in the stabilization of the posteromedial knee to the extent that PMC has been referred to as the “semimembranosus corner” [20]. SMt places traction on the posterior horn of the medial meniscus, preventing meniscal damage from compression between the femur and the tibia. In ACL-deficient knees, SMt limits anterior tibial translation during knee flexion by tightening POL and the posterior horn of the medial meniscus [18, 21]. SMt has a complex anatomy and past works provided variable descriptions of its insertions, but six main arms are consistently found in most studies [21, 22] (Supplemental Fig. 1). The OPL arm is the first detaching from the tendon and, as the name suggests, inserts into OPL, connecting SMt with PLC. The direct arm continues straight the course of the main tendon and has a wide insertion into the tuberculum tendinis of the posterior tibia. The anterior arm diverges anteriorly, running at first on the posterior aspect of the tibial component of POL and then inserting into the medial aspect of the tibial epiphysis [15]. The capsular arm arises from the distal tenosynovial sheath and inserts into the capsular component of POL. The meniscal arm connects the anterior arm with the coronary ligament of the medial meniscus [22]. Finally, the popliteal arm is an expansion of the direct arm that runs over the popliteus muscle). US allows a detailed evaluation of SMt and its expansions from the myotendinous junction to the distal insertions. (Supplemental Figs. 2 and 3). Overall, anatomic variations are common and may account for the numerous discrepancies in SMt descriptions found in previous studies (Supplemental Fig. 3). Recent US studies described thickening, hypoechogenicity, and loss of fibrillar pattern as hallmarks of partial tendon tears, which are usually associated with POL sprain [18, 23] (Fig. 1). In severe injuries avulsion of the tendon with bony detachment from the posteromedial tibia may occur often in association with ACL, MCL, and PCL tear [24–28]. Interestingly, a potential role of US in the diagnosis of unstable meniscocapsular separation through the dynamic evaluation of the meniscal arm of SMt during active knee flexion has been recently reported by some authors [29, 30]. However, it should be noted that to date no data are available on the diagnostic performance of US and MRI for SMt partial and complete tears.

### POL (ligament of Winslow)

**Fig. 2:**
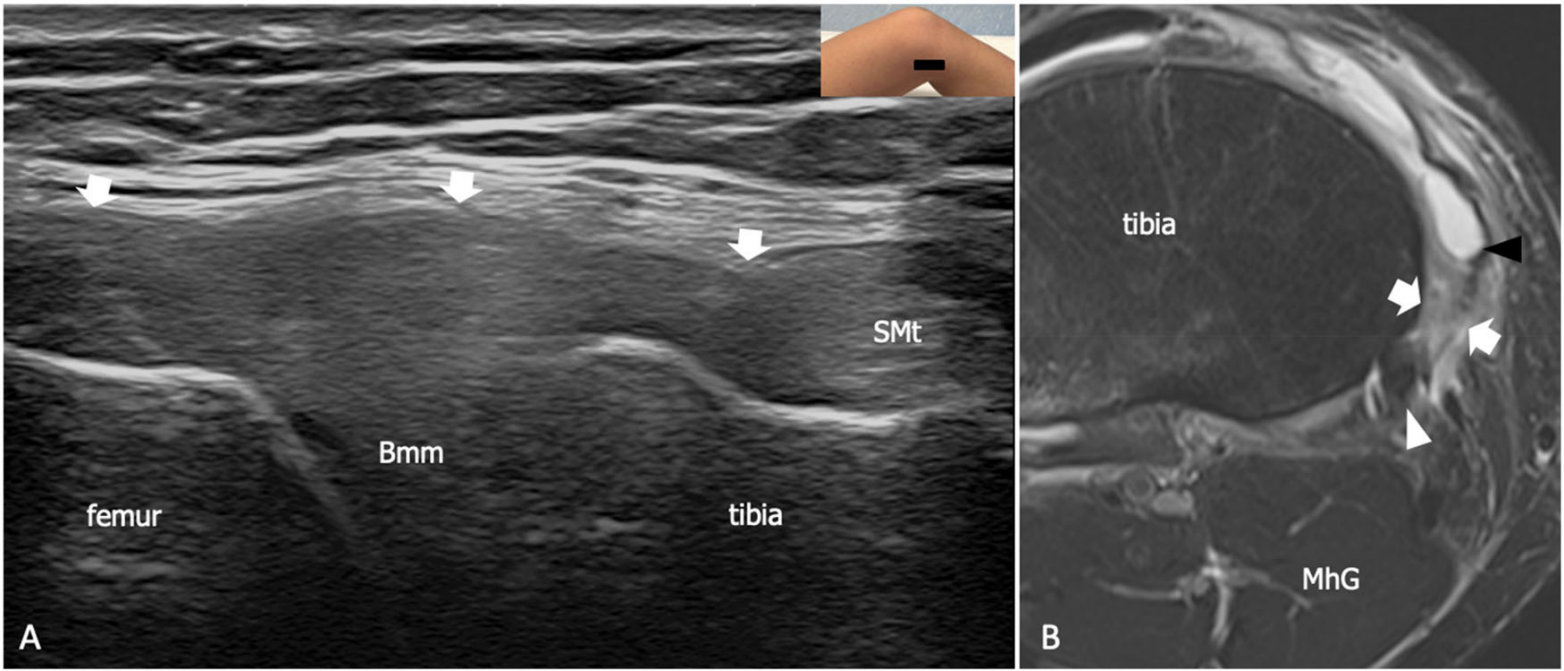
Partial tear of the superficial arm of POL in a 51-year-old woman with a recent knee sprain. **A** Long-axis 18-5 MHz US image and (**B**) comparative axial tSE T2-weighted 3 T scan with fat saturation shows irregular thickening and inhomogeneity of the superficial arm of POL (arrows). On US, the ligament is hypoechoic and does not appear fibrillar, whereas MRI demonstrates ligament swelling and interstitial edema. Note the coexistence of sprain of the anterior arm of SMt (Smt in **A**, white arrowhead in **B**) and of the medial collateral ligament (black arrowhead in **B**). Bmm, body of the medial meniscus. MhG, medial head of the gastrocnemius. The inserts illustrate the respective probe position

POL is a trapezoidal-shaped ligament with a narrow proximal origin close to the adductor tubercle and a wide insertion into SMt, the joint capsule, the medial meniscus, and the tibia (Supplemental Fig. 4) [31]. POL has a pivotal role in stabilizing the tibiofemoral joint against internal rotation in extension and early flexion angles and resists valgus stress and anteroposterior tibial translation [32, 33]. Its anterior margin is inseparable from the superficial MCL, whereas posteriorly the ligament blends with the joint capsule [21]. Three arms of POL have been described based on the different orientations of the ligament’s fibers. The superficial arm has a vertical course parallel and posterior to the superficial MCL and merges distally with the anterior arm of SMt. The tibial or central arm is the strongest and has an oblique course toward the posterior, inserting into the medial meniscus, the meniscofemoral and meniscotibial ligaments, and the tibia. In its distal part, the tibial arm is in close relationship with the anterior arm of SMt, which runs on its posterior aspect. The thin capsular arm connects the posteromedial capsule, the meniscofemoral ligament, and the medial part of OPL with the anterior tenosynovial sheath of SMt [34]. US allows for distinguishing the different components of POL based on their respective relationships with the superficial MCL and the anterior arm of SMt, and a potential role of this modality in diagnosing ligament tears has been suggested in past works, even if further research is needed to clarify its value (Supplemental Fig. 4) [23]. When the ligament is torn, US findings include thickening, interruption of fiber continuity, inhomogeneity, and loss of fibrillar pattern (Figs. 1 and 2). Comparison of the two sides may help in detecting subtle abnormalities such as mild ligament thickening in low-grade and chronic injuries, whereas dynamic evaluation during valgus stress increases the diagnostic confidence in identifying ligament laxity in unstable knees [35].

### OPL

**Fig. 3:**
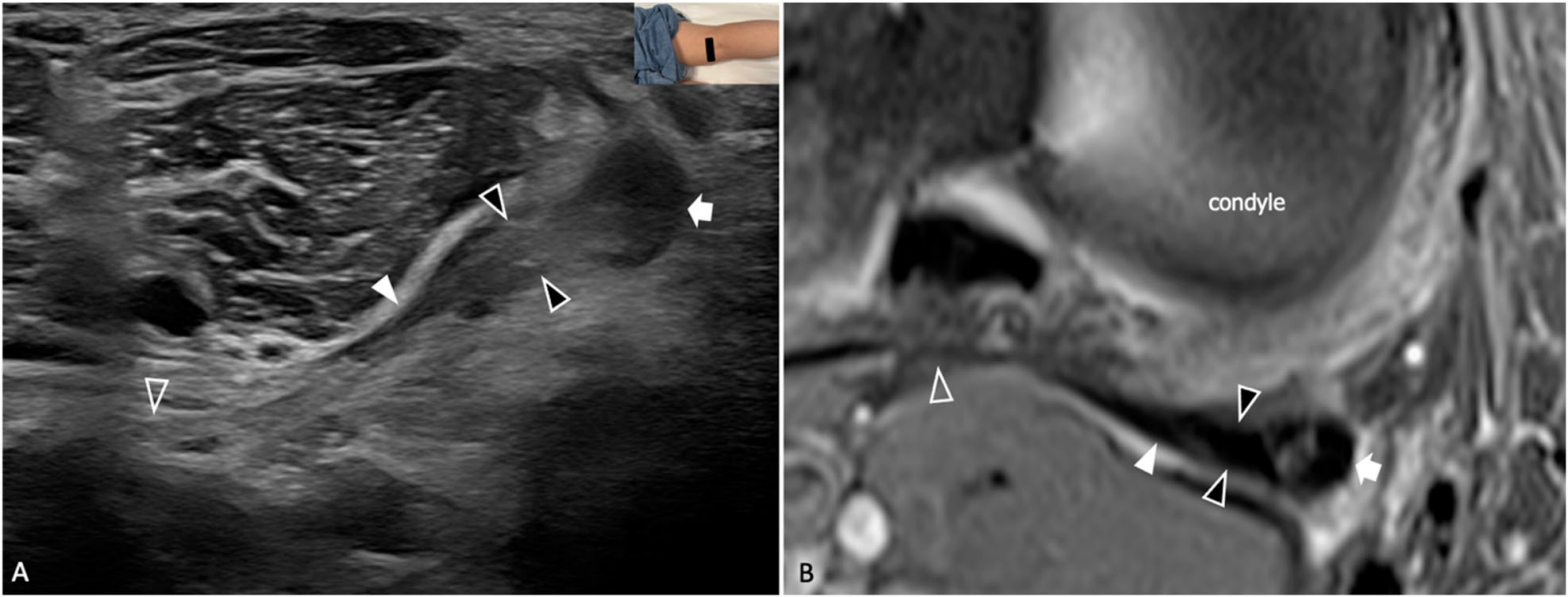
OPL injury in a 46-year-old amateur tennis player who suffered a knee sprain while quickly changing direction during a match six weeks before the exams. **A** Long-axis 18-5 MHz US image demonstrates thickening, hypoechogenicity, and loss of fibrillar structure of the oblique popliteal arm of SMt (black arrowheads) and OPL (white arrowhead), in relation to partial tears. Compare the abnormal echotexture of the origin of OPL with the regular appearance of its lateral part (outlined arrowhead). The main tendon of the semimembranosus also appears abnormally thickened on US (white arrow) (**B**) comparative axial tSE T2-weighted 3 T scan with fat saturation. After the resolution of edema, the oblique popliteal arm of SMt and OPL have a nearly regular appearance on MRI. Residual interstitial edema of the main tendon of the semimembranosus is demonstrated (white arrow), with hyperintensity on fluid-sensitive sequences. The inserts illustrate the respective probe position

OPL originates from the capsular arm of POL and the OPL expansion of SMt and inserts into the superolateral joint capsule and the fabella. Anatomical variants have been described in up to 70% of cases and include complex-shaped ligaments or the presence of more than one parallel bundle of fibers [36, 37]. OPL is a primary restraint to knee hyperflexion and plays a role in preventing excessive external rotation. OPL tears occur in high-energy knee sprains with multiligamentous injuries and have been linked to the development of posttraumatic genu recurvatum, a condition characterized by hyperextension and laxity of the tibiofemoral joint [36]. Currently, OPL injuries are underdiagnosed and managed conservatively, but the increasing awareness of their role in posterior knee stability has led several authors to advocate for a more precise diagnostic assessment after trauma [37]. Only a few reports exist on the US and MRI appearance of the normal and pathological OPL, and no data is currently available on the diagnostic accuracy of imaging modalities in the case of tear [14]. US allows to identify OPL as a fibrillar, transversely oriented structure originating from the OPL arm of SMt and running over the posterior aspect of the joint capsule [10] (Supplemental Fig. 2). In case of injury the ligament appears thickened and shows an inhomogeneous hypoechoic echotexture, findings which are consistently associated with tears of the OPL expansion of SMt (Fig. 3). In this context, attention should be paid in not misinterpreting for a tear the normal anisotropy of the medial third of the ligament related to its slight posteroanterior course; comparison between the two sides is mandatory to correctly diagnose doubtful cases.

### Clinically relevant stabilizers of the posterolateral corner (PLC)

The list of PLC stabilizers is so unsettled in the orthopedic, radiological, and anatomic literature that some authors referred to this area as ‘’the dark side of the knee” [38]. A major source of confusion is the existence of the so-called arcuate ligament, which has been variably described as an independent ligament, a complex formed by OPL and a thin accessory bundle of the fabellofibular ligament (FFL), or a structure formed by the popliteofibular ligament (PFL) and FFL [39, 40]. The prevalence and exact anatomy of FFL itself are a matter of debate, with visualization rates in anatomic specimens ranging from 20% to 87% (Supplemental Fig. 5) [41]. Overall, considering the frequency of injury and the amenability to surgical repair, the clinically relevant stabilizers of PLC include the lateral collateral ligament (LCL), the anterolateral ligament (ALL), and the popliteus muscle–tendon complex (Supplemental Fig. 6 and Table 2). Twisting, forced hyperextension, and direct blows on the anterior knee are listed amongst the mechanisms of injury of PLC. Only 28% of PLC injuries occur in isolation, whereas in most cases, they are associated with ACL tears [42]. In addition, stretching injuries of the peroneal nerve are reported in 12.7% of patients with PLC traumas [43]. Combined high-grade LCL and popliteus muscle–tendon complex injuries may lead to instability when the knee is in extension, including sensation of giving way during descending stairs, twisting, and pivoting, a condition which has been referred to as posterolateral rotatory instability (PLRI) [44]. Patient management varies according to the extent of damage, the presence of associated injuries, and clinical findings. In most instances, low-grade injuries can be managed conservatively with good functional results. High-grade PLC injuries have poor functional outcomes when managed non-operatively and should be addressed with anatomical reconstruction of the damaged structures. Finally, unrecognized and untreated PLC tears have been linked to poor postoperative results after ACL reconstruction [42, 43, 45].

**Table 2:** PLC stabilizers

	Normal US appearance	Pathological findings
Lateral collateral ligament	Hyperechoic and fibrillar. Slight hypoechogenicity around the proximal insertion due to mucin content	Thickening and inhomogeneous echotexture in partial tears; interruption of fibers’ continuity and wavy appearance in severe traumas
ALL	Thin fibrillar expansion of the tibiofemoral joint capsule inserting into the tibial epiphysis posterior to the iliotibial band	Thickening, wavy appearance, and bone avulsion from the distal insertion
Popliteus tendon	Short, thick, and fibrillar tendon with a curved course inserting into the popliteal notch	Hypoechoic thickening in mild and moderate strain, detachment from bone in severe trauma
PFL	Fibrillar band connecting the popliteus myotendinous junction with the fibular head, deep to the inferior genicular vessels	Thickening and hypoechogenic appearance

### Lateral collateral ligament

LCL is an extrarticular ligament that originates from the lateral epicondyle and inserts on the fibular head, with a length of approximately 7 cm [46]. LCL is the most important stabilizer against varus forces, but also has a significant role in preventing rotational instability [47]. LCL damage has been reported in 4% of high-energy knee trauma, representing the second least common knee ligament tear after PCL injuries, and is usually found in complex, multiligamentous traumas [48, 49]. US findings in mild and moderate injuries include thickening, hypoechogenicity, and inhomogeneous echotexture, whereas avulsion and interruption of fibers continuity are indicative of severe traumas (Supplemental Fig. 7). In a previous study, US showed a sensitivity, specificity, negative predictive value, positive predictive value, and accuracy of 92%, 75%, 92%, 75%, and 88%, respectively for diagnosing LCL tears using surgical exploration as reference standard [50]. In the same work, dynamic assessment of joint stability with US increased the diagnostic accuracy for detecting LCL and PLC injuries, as sonographic evidence of a lateral femoro-tibial joint space exceeding 10.5 mm during varus stress demonstrated a sensitivity of 83% and a specificity of 100%. This corresponded to a positive predictive value of 100%, a negative predictive value of 75%, and an overall diagnostic accuracy of 88%. Pathological findings are often more severe at the proximal third of the ligament, but slight hypoechogenicity of LCL in proximity to its femoral insertion should not be misinterpreted as low-grade tears, as this finding may be related to a non-pathological increase in mucin content inside the matrix of the ligament [51]. Comparison between the two sides may help in solving doubtful cases.

### ALL

**Fig. 4:**
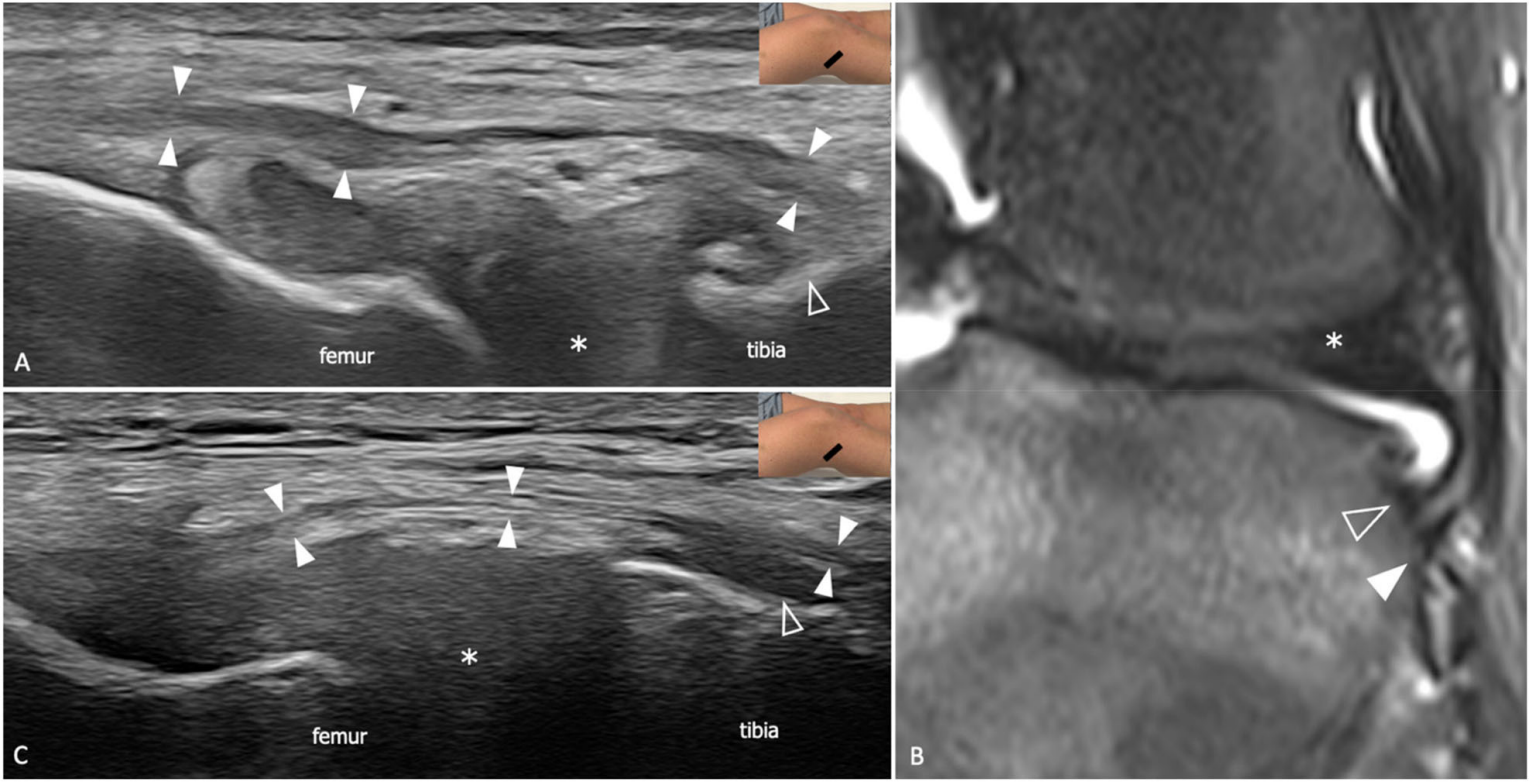
ALL tear in a 31-year-old male patient with a recent knee sprain and full-thickness ACL tear. **A** Longitudinal 22-8 MHz US image and (**B**) coronal fat-saturated tSE T2-weighted 3 T scan show thickening and wavy appearance of ALL (arrowheads). Note the lateral meniscotibial ligament (outlined arrowhead) inserting into the tibial plateau in close proximity to ALL. **C** Longitudinal 22-8 MHz US from the contralateral knee of the same patient demonstrates the normal appearance of ALL (arrowheads). Outlined arrowhead, meniscotibial ligament; asterisk, meniscal body. The inserts illustrate the respective probe position

An expansion of the lateral tibiofemoral joint capsule connecting the femoral condyle with the anterolateral aspect of the proximal tibia has been recognized in several past dissection works and sometimes has been included amongst PLC stabilizers with variable descriptions and nomenclature, including ALL, lateral capsular ligament, and mid-third lateral capsular ligament [52]. ALL strain has been linked to the avulsion of a small piece of bone from the tibial epiphysis, which has been referred to as Segond’s fracture, but its clinical relevance lies in its role in the persistence of knee instability after reconstruction of torn ACL [53, 54]. ALL originates from the lateral epicondyle of the femur, slightly posterior to the proximal insertions of LCL. Above the tibiofemoral joint line, it courses almost parallel to LCL, but then diverges anteriorly to insert into the proximal tibia between Gerdy’s tubercle and the fibular head [55, 56]. The US can depict ALL in up to 93.8% of subjects with a strong interobserver agreement for thickness and length measurements [57]. US findings consistent with ALL injuries have been described in 53% of patients with suspected ACL tear [58, 59]. Wavy appearance and ligament thickening are the hallmarks of ALL disruption and may be associated with avulsion of a small flake of bone from the tibial insertion (Fig. 4) [60].

### Popliteus muscle–tendon complex

**Fig. 5:**
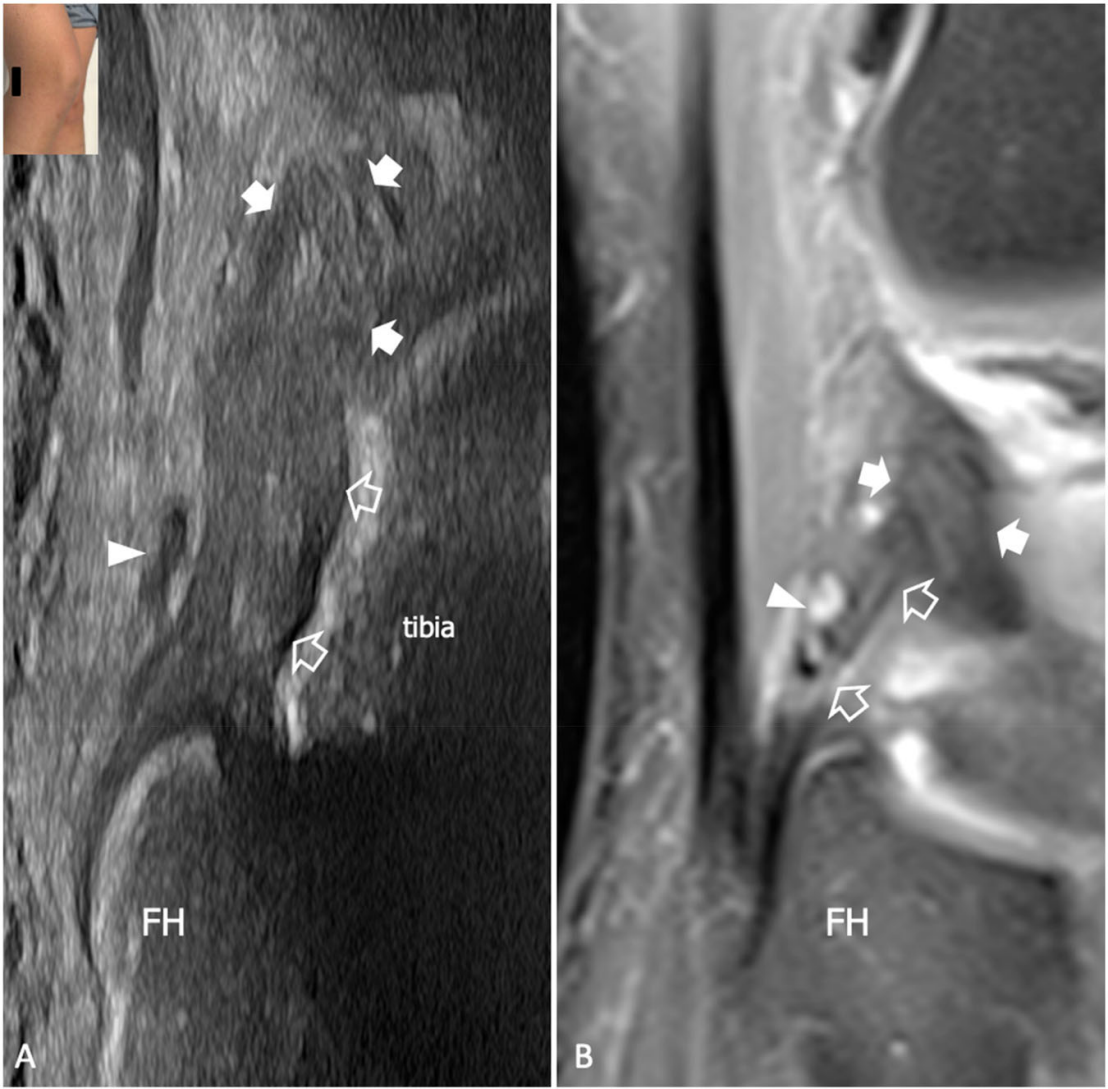
PFL tear in a 58-year-old man who suffered a recent knee trauma with partial tear of ACL and longitudinal tear of the medial meniscus. **A** Long-axis 18-5 MHz US image and (**B**) comparative coronal fat-saturated tSE T2-weighted 3 T scan show thickening and inhomogeneous appearance of PFL (outlined arrows). On MRI, the ligament is mildly hyperintense, but overall, abnormal findings are more evident on US. Note the anatomic position of the ligament, which connects the popliteus tendon (arrows) and the fibular head (FH), running deep to the inferolateral genicular vessels (arrowhead). The inserts illustrate the respective probe position

**Fig. 6:**
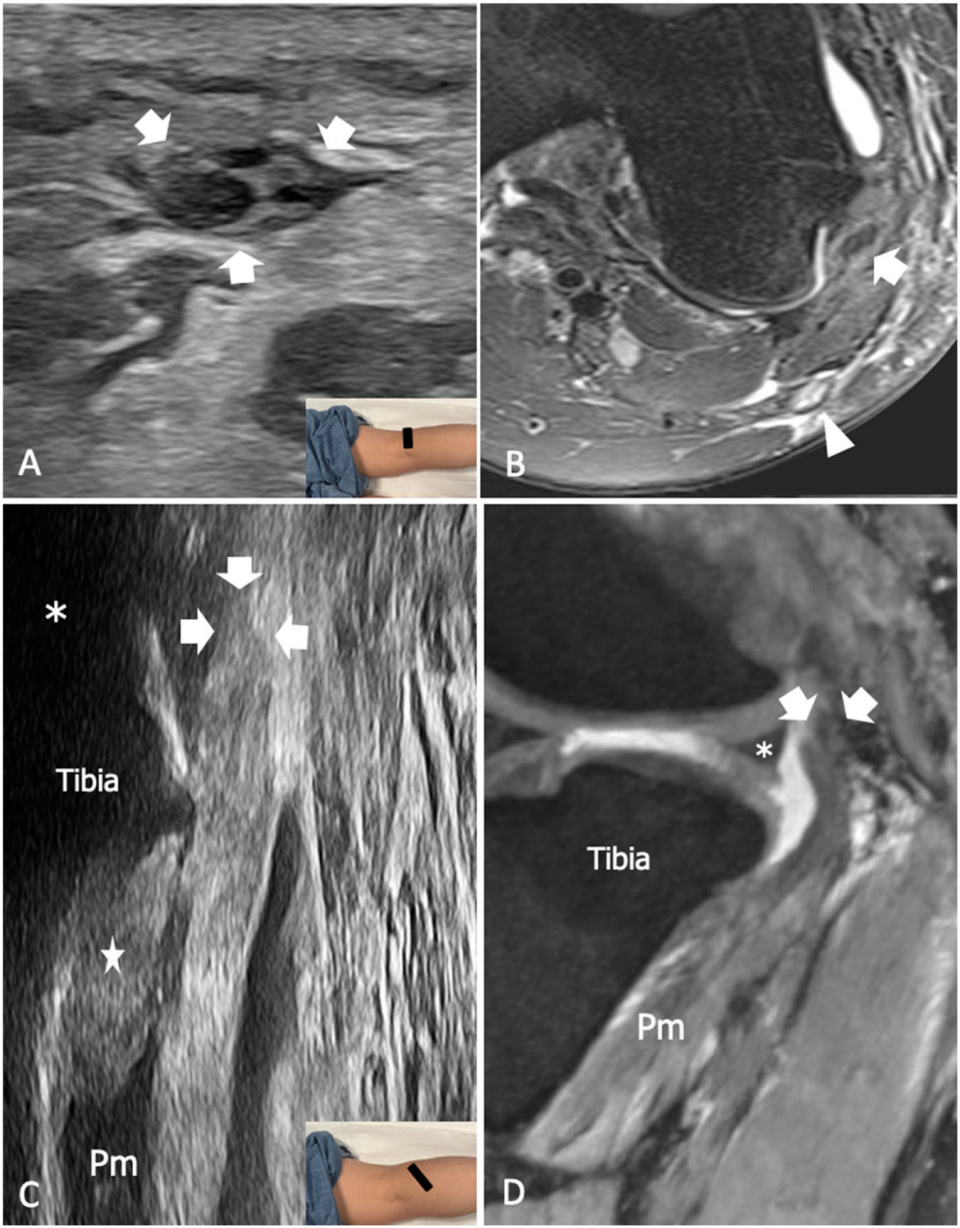
High-grade injury of PLC following a motorcycle accident in a 33-year-old patient who complained of knee pain and motor and sensory symptoms in the territory of the common peroneal nerve. **A** Short-axis 24-9 MHz US image demonstrates thickening and intrafascicular edema of the common peroneal nerve along its course across the popliteal fossa. **B** Axial fat-saturated tSE T2-weighted 3 T scan confirms fascicular edema with hyperintensity on fluid-sensitive sequences of the common peroneal nerve (arrowhead). LCL (arrow) has an inhomogeneous and hyperintense appearance in relation to a sprain injury (**C**) longitudinal 18-5 MHz US image demonstrates a full-thickness tear of the popliteus tendon with retraction of the proximal stump (arrows) at the level of the joint line (asterisk). Hyperechoic areas (star) are demonstrated inside the belly of the popliteus (Pm) in relation to intramuscular edema and hemorrhage. **D** Oblique MPR double echo steady state (DESS) MR scan confirms the avulsion of the popliteus tendon with the proximal stump (arrows) in close proximity to the lateral meniscus (asterisk), as well as the edema of the popliteus muscle (Pm). The inserts illustrate the respective probe position

The popliteus muscle originates from the posterior surface of the proximal tibia and inserts through a short tendon on the popliteal notch of the lateral femoral condyle. Inside the notch, the tendon is in an intracapsular and extrasynovial position, whereas more distally it pierces the tibiofemoral joint capsule, passing through a fibrous tunnel referred to as the popliteal hiatus. Despite variable anatomic descriptions and nomenclature, multiple popliteomeniscal bands can be recognized connecting the popliteus muscle and tendon to the body and the posterior horn of the lateral meniscus [61]. Medial to the hiatus, PFL is a critical PLC stabilizer that secures the popliteus myotendinous junction to the styloid process of the fibula. The ligament is present in 88.3% of subjects and in most cases consists of a single band, even if variations have been described [62]. Overall, biomechanical studies showed that the popliteus tendon and PFL are the main stabilizers of the knee against external rotation at 60 degrees and 90 degrees of knee flexion, while LCL has higher load sharing with the knee in extension [63]. When combined with LCL insufficiency, high-grade injuries of the popliteus muscle–tendon complex may result in severe knee instability and require surgical repair [64]. US can show the popliteus in the popliteal fossa as a fusiform transversely-oriented muscle originating from the posterior proximal tibia and coursing on the anterior aspect of the popliteal artery and vein [65]. Around the myotendinous junction, PFL is readily identified as a discrete fibrillar structure connecting the popliteus myotendinous junction with the fibular head, deep to the inferolateral genicular vessel [12]. In some cases, short fibrillar bands can be demonstrated bridging between the popliteus tendon and the meniscus, representing the popliteomeniscal ligaments. Finally, the popliteus tendon can be seen inserting into the condyle at the level of the popliteal notch (Supplemental Fig. 8). In this area, the tenosynovial sheath is in continuity with the joint cavity; effusion around the tendon may not be indicative of tendinopathy. Strain of PFL is not uncommonly found in patients with ACL tears and may be associated with LCL injury or interstitial edema of the popliteus muscle (Fig. 5). US can demonstrate PFL lesion with 67% sensitivity, 75% specificity, 67% negative predictive value, 75% positive predictive value, and 69% accuracy [50]. Injuries of the popliteus more often involve the muscle belly or the myotendinous junction [65]. In this context, imaging findings include intramuscular edema or detachment of muscle fibers from the intramuscular aponeurosis with formation of hematoma (Fig. 6C). US findings in partial tear of the popliteus tendon have been described and include thickening, hypoechogenicity, and loss of fibrillar echotexture [66]. Detachment of the popliteus tendon from the condyle is rare and may be seen in severe trauma where it is usually associated with damage of other stabilizers of PLC, cruciate ligament tear, and peroneal neuropathy (Fig. 6). Due to the rarity of these injuries, the diagnostic performance of US and MRI in detecting partial and complete tear of the popliteus has yet to be addressed.

## Conclusions

High-resolution US allows an in-depth evaluation of the clinically relevant stabilizers of PMC and PLC and can integrate clinical and MRI findings, providing an accurate picture of the structures involved by the trauma. In subacute and chronic traumas, US may disclose subtle findings indicative of PMC/PLC damage that may be missed on MRI.

## Supplementary information

ELECTRONIC SUPPLEMENTARY MATERIAL

## Abbreviations

ACL: Anterior cruciate ligament
ALL: Anterolateral ligament
FFL: Fabellofibular ligament
OPL: Oblique popliteal ligament
PFL: Popliteofibular ligament
PLC: Posterolateral corner
PMC: Posteromedial corner
POL: Posterior oblique ligament
SMt: Semimembranosus tendon
