# Supplementary material for: Clinically relevant stabilizers of the posteromedial and posterolateral knee: normal anatomy, scanning technique, and ultrasound findings in patients with anterior cruciate ligament tear
Source: Eur Radiol. 2025 Jul 29;36(1):504–14. doi: 10.1007/s00330-025-11868-8 (PMC12712078; doi:10.1007/s00330-025-11868-8)
Supplement: Supplementary file 1 — ELECTRONIC SUPPLEMENTARY MATERIAL [file 330_2025_11868_MOESM1_ESM.pdf]

Clinically relevant stabilizers of the posteromedial and posterolateral knee: normal anatomy, scanning technique, and ultrasound findings in patients with anterior cruciate ligament tear

ELECTRONIC SUPPLEMENTARY MATERIAL

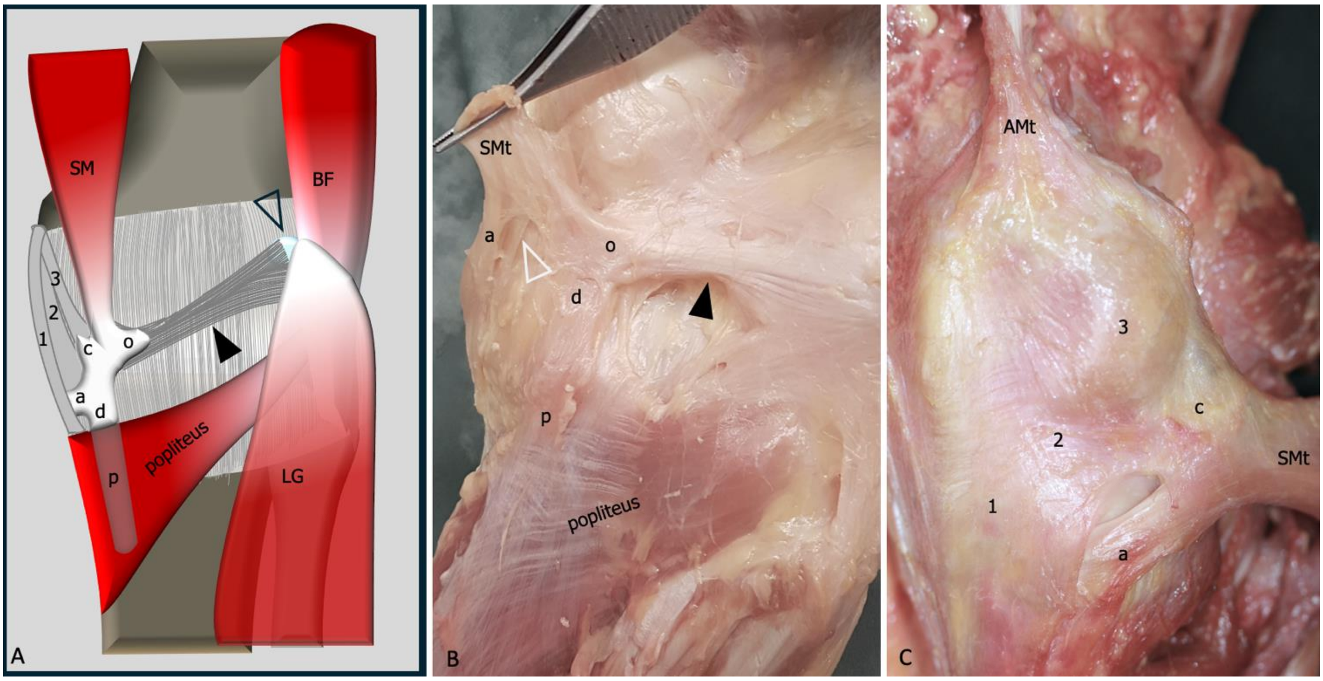

Supplemental Figure 1: Semimembranosus tendon anatomy. A) Schematic drawing and B,C) Cadaveric specimens illustrate the direct (d), anterior (a), capsular (c), oblique popliteal (o), popliteal, and meniscal (white outlined arrowhead) arms of the semimembranosus tendon (SMt). Black arrowhead, oblique popliteal ligament; black outlined arrowhead, fabella; SM, semimembranosus muscle; BF, bicep femoris; LG, lateral gastrocnemius; 1 superficial arm of the posterior oblique ligament; 2, tibial arm of the posterior oblique ligament; 3, capsular arm of the posterior oblique ligament. AMt, adductor magnus tendon.

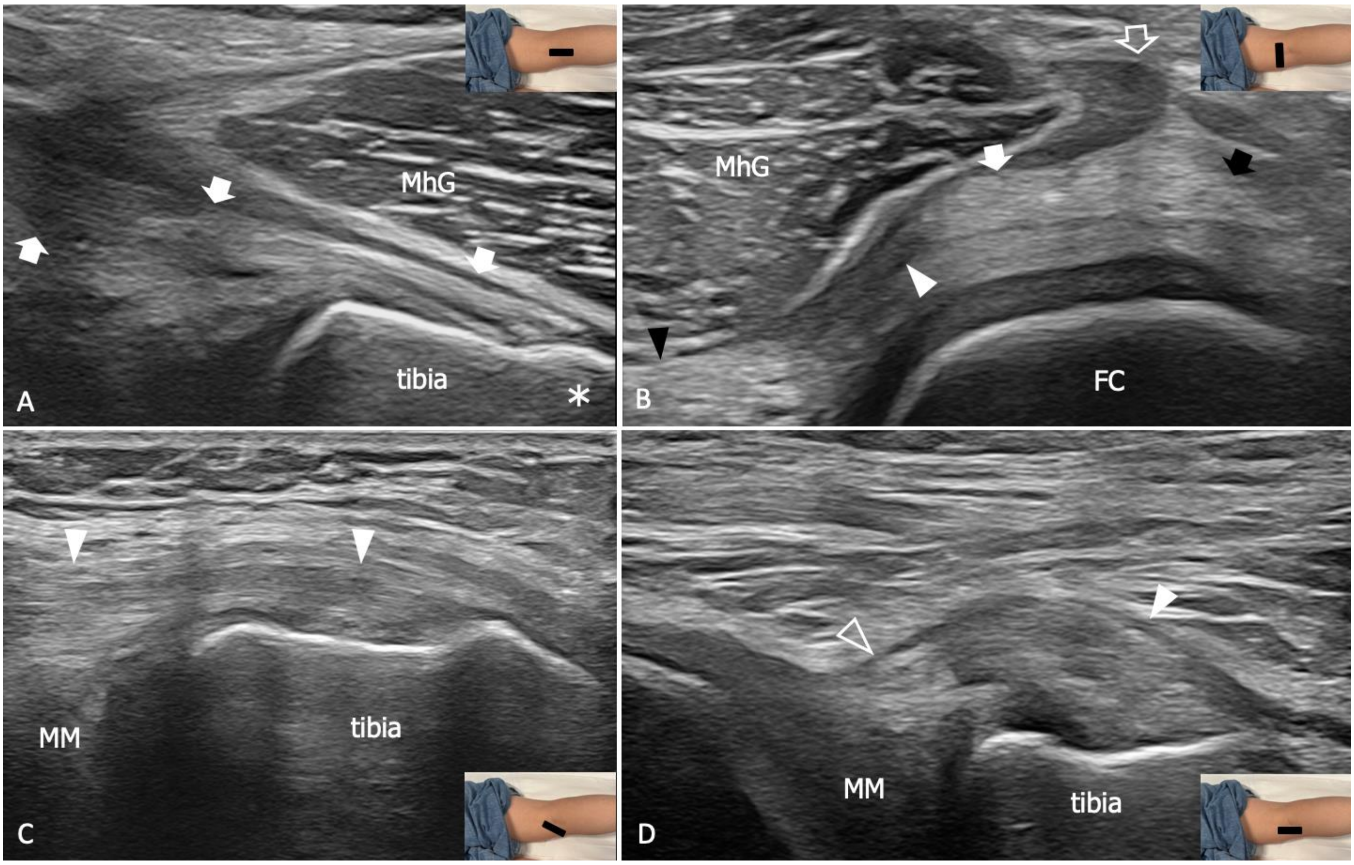

Supplemental Figure 2: Semimembranosus tendon anatomy. A) Longitudinal 18-5MHz US image shows the direct arm (arrows) inserting on the tuberculum tendinis of the tibia (asterisk). B) Transverse 18-5MHz US image shows the anterior (black arrow), direct (white arrow), and oblique popliteal arms (white arrowhead) detaching from the distal tendon of the semimembranosus. The oblique popliteal ligament (black arrowhead) originates from the oblique popliteal arm of the semimembranosus tendon and runs towards the posterolateral corner. Outlined arrow, medial head of the gastrocnemius tendon. C) Longitudinal 18-5MHz US image demonstrates the insertion of the anterior arm (arrowheads). D) Oblique 18-5MHz US image illustrates the meniscal expansion (outlined arrowheads) detaching from the anterior arm (arrowhead) and inserting into the medial meniscus (MM). MhG, medial head of the gastrocnemius muscle. The inserts illustrate the respective probe position.

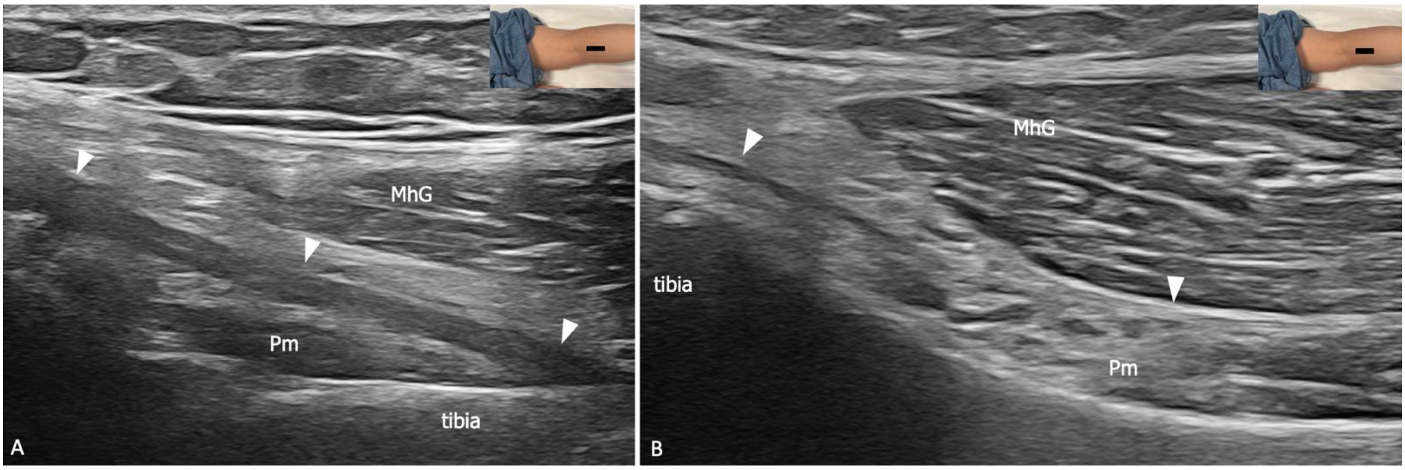

Supplemental Figure 3: Semimembranosus tendon variation. A) Longitudinal 18-5MHz US image shows an abnormally thickened and cord-like popliteal expansion of the semimembranosus tendon (arrowheads) running over the popliteus muscle (Pm) and inserting in the proximal third of the tibial diaphysis. B) Longitudinal 18-5MHz US image obtained in another subject demonstrates the normal appearance of the popliteal arm, which consists in a thin expansion covering the popliteus muscle. MhG, medial head of the gastrocnemius. The inserts illustrate the respective probe position.

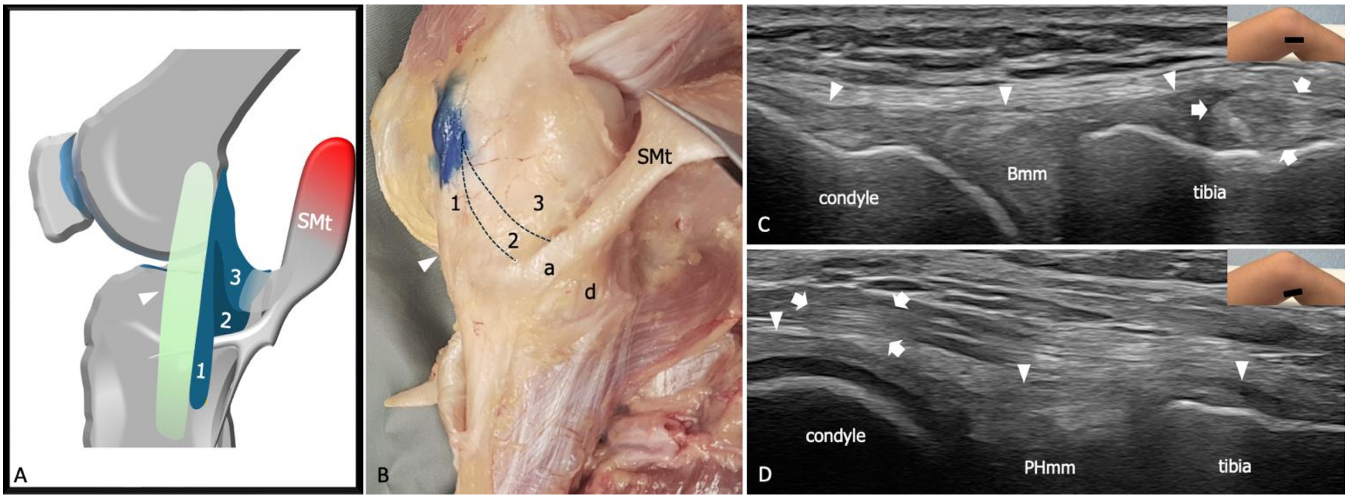

Supplemental Figure 4: Posterior oblique ligament. A) Schematic drawing and B) Cadaveric specimen illustrate the superficial (1), tibial (2), and capsular arms (3) of the posterior oblique ligament and their respective relationship with the superficial medial collateral ligament (arrowhead) and the anterior (a) and the direct (d) arms of the semimembranosus tendon (SMt). Blue stain marks the adductor tubercle. C) Long-axis 18-5MHz US image demonstrates the superficial arm (arrowheads) running over the joint line and the body of the medial meniscus (Bmm) and merging with the anterior arm of the semimebranosus tendon (arrows) in proximity to its distal insertion on the tibia. D) Long-axis 18-5MHz US image shows the relationship of the tibial arm of the posterior oblique ligament (arrowhead) with the anterior arm of the semimembranosus (arrows) and the posterior horn of the medial meniscus (PHmm). The inserts illustrate the respective probe position.

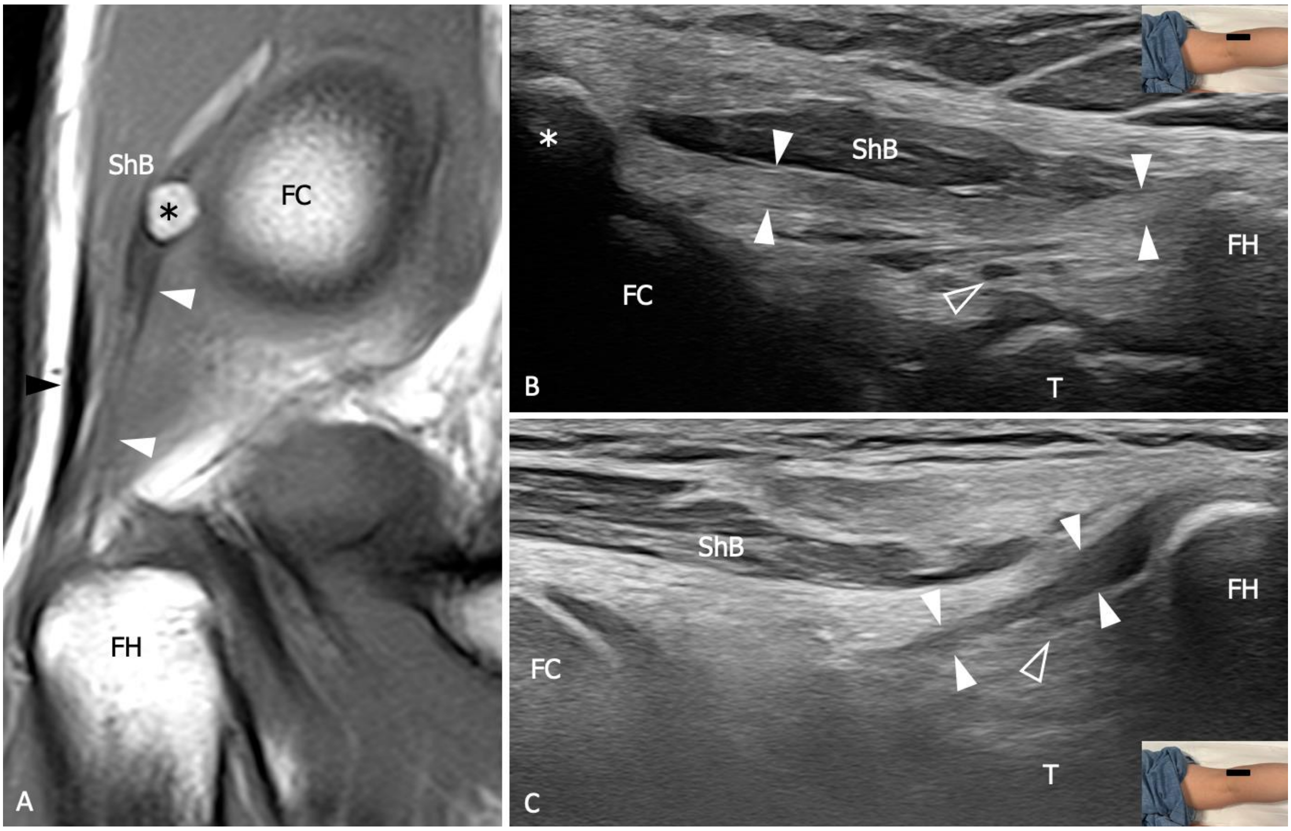

Supplemental Figure 5: Fabellofibular ligament anatomy. A) Coronal tSE T1-weighted MRI scan and (B) Long-axis 18-5MHz US image shows the fabellofibular ligament (arrowheads) connecting the fibular head (FH) with the fabella (asterisk). Note the position of the ligament relative to the short head of the bicep muscle (ShB) and the inferolateral genicular vessels (outlined arrowhead). Black arrowhead, distal tendon of the bicep femoris. (C) Long-axis 18-5MHz US image shows an anatomical variant in a patient without a fabella, in whom an anomalous fabellofibular ligament (arrowheads) has a short and oblique course and inserts directly into the joint capsule. Despite the abnormal course, the ligament maintains its regular position relative to the inferolateral genicular vessels (outlined arrowhead), which are typically used as a landmark for its identification in anatomic works. FC, femoral condyle; T, tibia. The inserts illustrate the respective probe position.

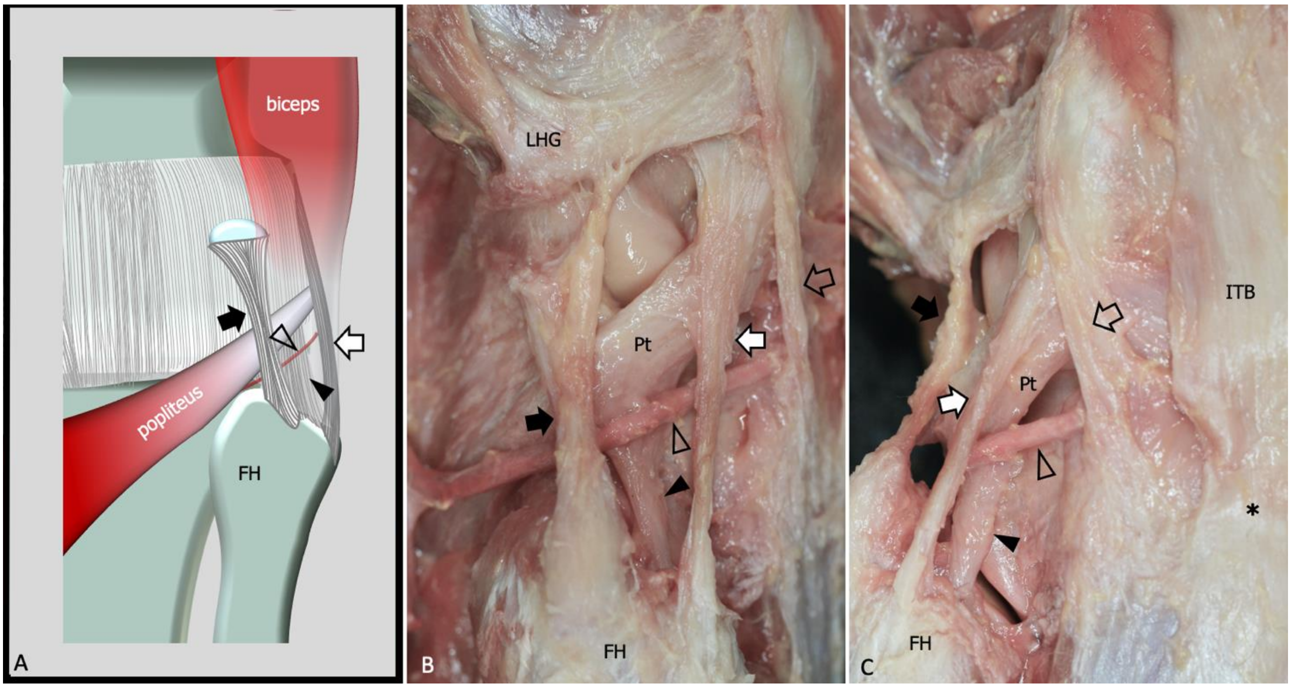

Supplemental Figure 6: Posterolateral corner of the knee. A) Schematic drawing and (B,C) Anatomic dissections show the fabellofibular (black arrow), lateral collateral (white arrow), popliteofibular (black arrowhead), and anterolateral (outlined arrow) ligaments. Note the inferolateral genicular artery (outlined arrowhead) running between the fabellofibular and the popliteofibular ligaments. FH, fibular head; Pt, popliteus tendon; ITB, iliotibial band; asterisk, Gerdy's tubercle.

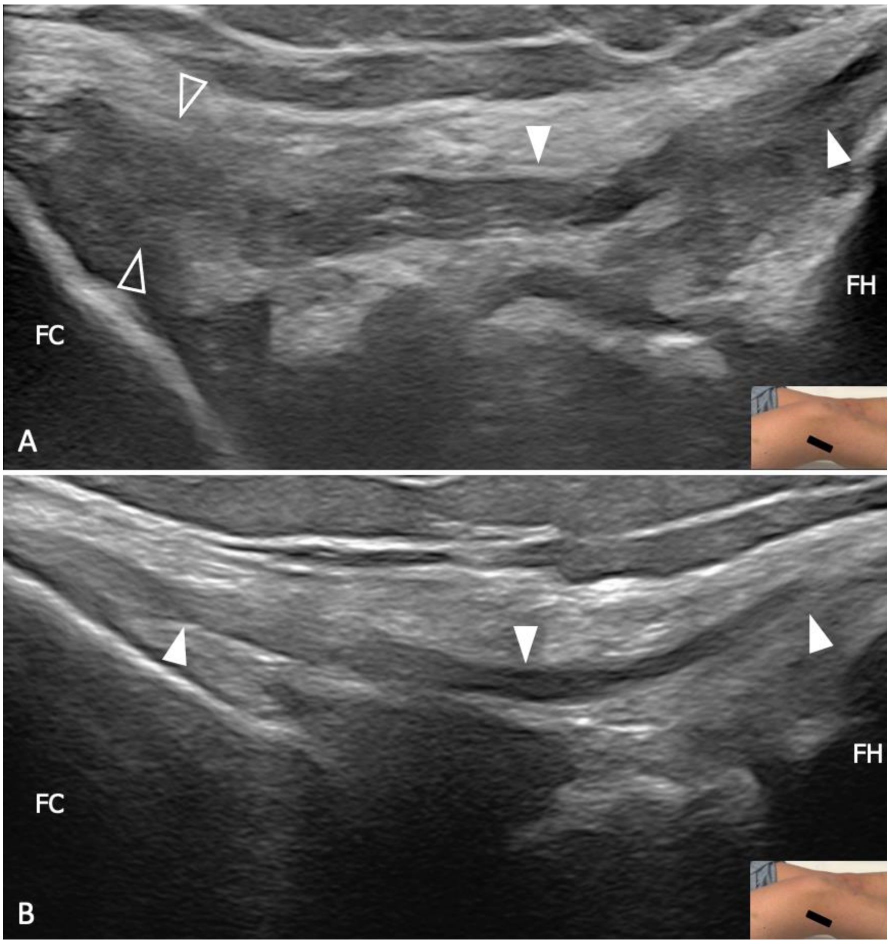

Supplemental Figure 7: Partial tear of the lateral collateral ligament in a 56yo amateur football player. A) Long-axis 18-5MHz US demonstrates thickening, hypoechogeneity, and loss of fibrillar structure of the lateral collateral ligament (arrowheads). Note that pathological findings are more severe at the proximal third of the ligament (outlined arrowhead). B) Long-axis 18-5MHz US of the contralateral knee shows the normal appearance of the ligament (arrowheads). The inserts illustrate the respective probe position.

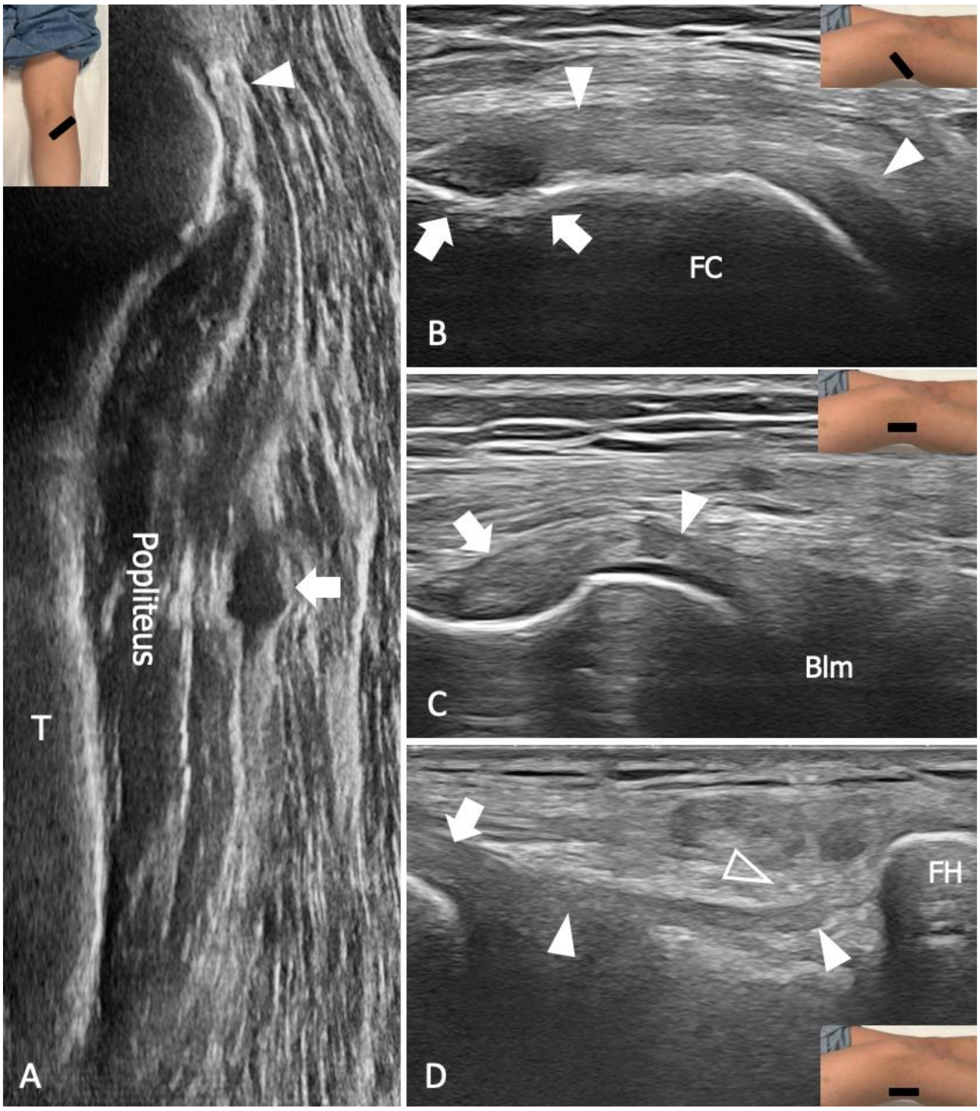

Supplemental Figure 8: Popliteus muscle-tendon complex. A) Longitudinal 18-5MHz US image shows the popliteus muscle originating from the tibial epiphysis (T) and running on the anterior aspect of the popliteal artery (arrow). Arrowhead, popliteus tendon. B) Longitudinal 18-5MHz US scan demonstrates the popliteus tendon (arrowheads) inserting on the popliteal groove (arrows) on the lateral femoral condyle (FC). C) Short-axis 18-5MHz US image shows a popliteomeniscal band (arrowhead) connecting the popliteus tendon (arrow) with the body of the lateral meniscus. D) Longitudinal 18-5MHz US scan show the popliteofibular ligament (arrowheads) connecting the fibular head (FH) and the popliteus tendon (arrow), running on the anterior aspect of the inferolateral genicular vessels (outlined arrowhead). The inserts illustrate the respective probe position.
